# Re-examining survivorship research for people living with advanced and metastatic cancers: A portfolio analysis update of National Institutes of Health-funded grants

**DOI:** 10.1007/s11764-025-01935-w

**Published:** 2025-12-03

**Authors:** Michelle A. Mollica, Gina Tesauro, Emily S. Tonorezos, Lisa Gallicchio

**Affiliations:** 1 Division of Cancer Control and Population Sciences, National Cancer Institute, Bethesda, MD USA; 2 College of Nursing, Medical University of South Carolina, Charleston, SC USA; 3 Division of Cancer Prevention and Control, Hollings Cancer Center, Medical University of South Carolina, Charleston, SC USA; 4 Division of Hematology/Oncology, Department of Medicine, Weill Cornell Medical College, New York, NY USA

**Keywords:** Survivor, Metastatic, Advanced cancer, Cancer survivorship

## Abstract

**Purpose:**

The National Cancer Institute (NCI) identified survivorship research for people living with advanced and metastatic cancers as a priority in 2020. The goal of this portfolio analysis was to review all National Institutes of Health (NIH) grants newly funded in Fiscal Year (FY) 2021 to FY 2024 focused on this area.

**Methods:**

Grants were identified using a text mining algorithm of survivorship-relevant terms from the NIH Research, Condition, and Disease Categorization (RCDC) system and double coded for grant characteristics (e.g., study design, cancer type, primary focus, and primary outcomes).

**Results:**

Included in this analysis were 63 grants, funded by 7 NIH institutes. The number of newly awarded grants funded rose from 9 in 2021 to 25 in 2024. The majority of grants were R01s (62.9%). Cancer types most often included were breast (22.2%), prostate (15.9%), and lung (15.9%). The primary focus of studies was most often acute toxicities (33.3%) or late- or long-term effects (33.3%). No grants focused on the financial impact of an advanced or metastatic cancer diagnosis.

**Conclusions:**

Results indicate a substantial growth in the number of grants funded from FY 2021 to FY 2024. Grants address some of the gap areas identified in previous NCI efforts, including longitudinal studies of symptoms. Scientific gap areas that persist include financial hardship and employment, models of care delivery, and prognostic uncertainty.

**Implications for Cancer Survivors:**

Despite the growth in the grant portfolio, more research on survivors living with advanced and metastatic cancers is needed. This emerging population has unmet and understudied needs that will only expand given the growth in new therapies and targeted treatments.

## Introduction

Of the estimated 18.6 million cancer survivors in the United States [1], an increasing number are people living with cancer that is advanced (i.e., unlikely to be cured [2]) or metastatic. It is estimated that over 693,000 people are living with the most prevalent metastatic cancers in the US [3]. As people live longer with advanced and metastatic cancers, they often have emerging and unmet survivorship needs, including physical and psychosocial symptoms, financial hardship, caregiver burden, and great prognostic uncertainty [4–6]. Yet, these needs have not yet been systematically evaluated and addressed.

In 2020, an NIH-wide grant portfolio analysis conducted by Mollica and colleagues reviewed active grants receiving funding in Fiscal Year (FY) 2020 that were focused on people living with advanced and metastatic cancer [7]. Findings of that analysis revealed that most grants in this population were aimed at examining palliative care and psychosocial support. In response, the National Cancer Institute (NCI), in 2021, held a scientific meeting to convene subject matter experts, researchers, clinicians, survivors, and advocates to discuss the scientific gaps and opportunities in several areas: epidemiology and surveillance, symptom management, psychosocial research, healthcare delivery, and health behaviors [8]. A subsequent Request for Applications (RFA), *RFA-CA-22–027: Research to Understand and Address the Survivorship Needs of Individuals Living with Advanced Cancer*, was issued to solicit applications for observational and intervention studies aiming to better understand and/or address the needs of these individuals [9]. Through this RFA, 8 grants were funded that span multiple cancer types and focus on the breadth of needs for these survivors [10]. A subsequent Notice of Special Interest (NOSI) was published by the NCI in 2024 on this topic [11].

The purpose of this paper was to review research funded in response to NCI efforts to grow this portfolio. This analysis serves as an update to the 2020 analysis of currently funded grants and describes grants newly funded from FY 2021 to FY 2024 that focus on individuals living with advanced and metastatic cancers.

## Methods

This portfolio analysis used data from the NIH cancer survivorship research portfolio of newly funded grants from FY 2021–2024. The search strategy and definitions to identify survivorship research have been described elsewhere [12]. Briefly, NIH research grants (R series, U01s, and P01s) [13] were identified using a text mining algorithm of words from the NIH Research, Condition, and Disease Categorization (RCDC) thesaurus with survivorship-relevant terms (e.g., survivor, symptoms, quality of life). Two coauthors reviewed the title, abstract, and specific aims to determine inclusion in the broader survivorship portfolio. Grant information (e.g., NIH Institute, funding mechanism, notice of funding opportunity [NOFO]) was extracted. Study characteristics (e.g., study design, cancer type, primary focus, primary outcomes) were then double coded. Disagreements in coding were resolved with discussion.

For this analysis, grants that focused on people living with advanced and metastatic cancers were selected. Given the primary focus was understanding NIH-funded research exclusively on survivorship for individuals living with advanced or metastatic disease, grants that focused on people with early-stage cancer or included any cancer stage were excluded. Additionally, grants focused on end-of-life care, tumor progression or staging, as well as grants for which overall survival was the only outcome were excluded. Descriptive statistics were used to examine frequencies.

## Results

**Fig. 1:**
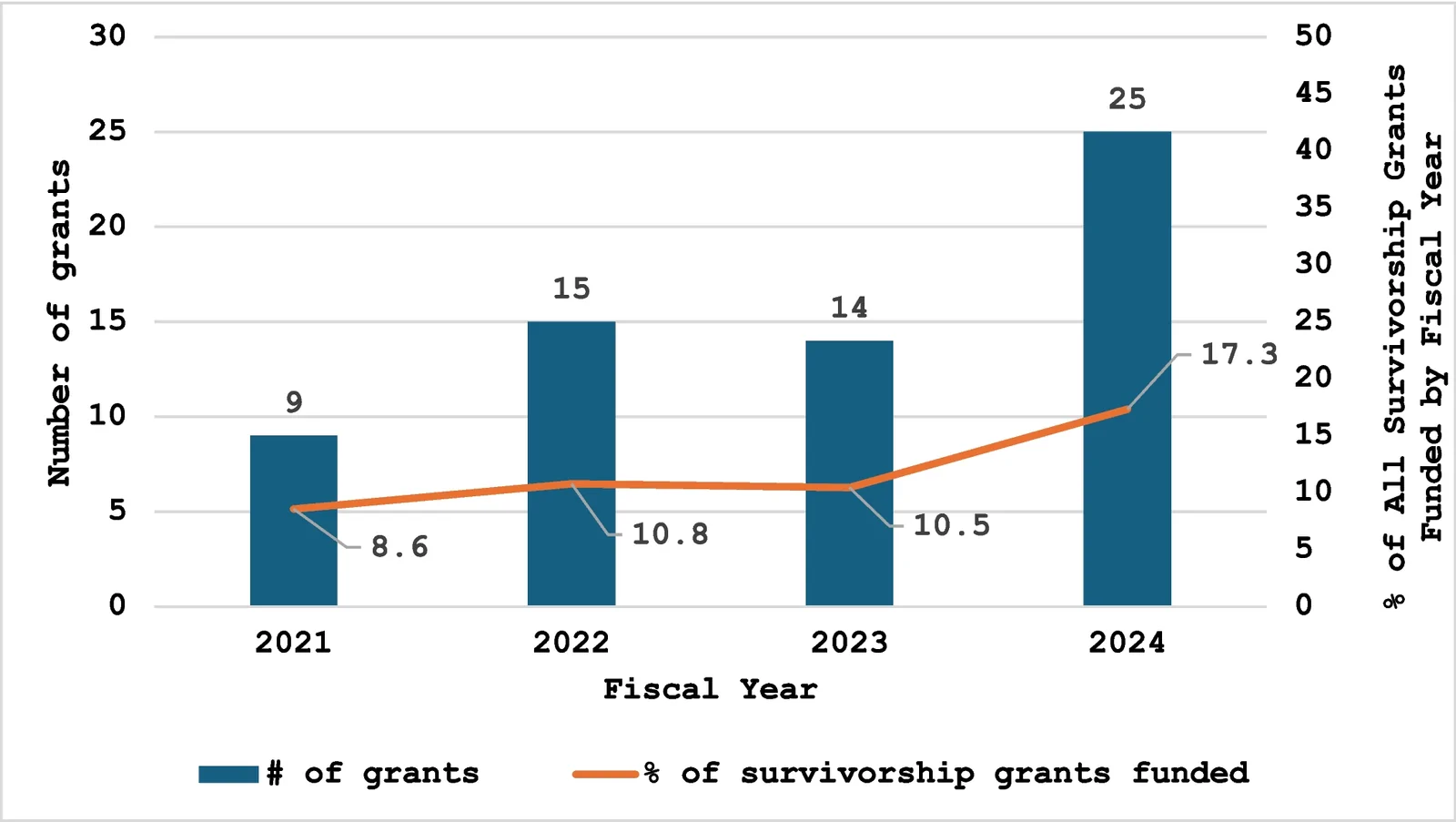
Number of Grants Funded by Fiscal Year and Percentage of All Survivorship Grants Funded (*N* = 63)

A total of 63 grants were included in this review, newly funded between FY 2021 to FY 2024. Overall, the number of grants funded each FY increased from 9 grants in 2021 to 25 grants in 2024 (Fig. 1; depicting number of grants funded per year and the percentage of grants focused on advanced and metastatic survivorship within the larger survivorship portfolio). Grants were funded by seven NIH Institutes, with the majority funded by NCI (85.7%) (Table 1). The R01 (61.9%) and R21 (20.6%) mechanisms were most common; a little over half of the grants (50.8%) were submitted through investigator-initiated funding opportunities (e.g. Cancer Prevention and Control Clinical Trials Grant Program - R01 Clinical Trial Required). Approximately 43% of grants were multiple PI (MPI) grants and of 43 eligible grants, 16.3% were led by Early-Stage Investigators (ESIs).

**Table 1:** Grant Characteristics (*N* = 63 grants)

Characteristic	*N*	%
**NIH Institute **		
National Cancer Institute	54	85.7
National Institute on Aging	2	3.2
National Institute of Nursing Research	4	6.3
National Institute of Arthritis and Musculoskeletal and Skin Diseases	1	1.6
National Institute on Minority Health and Health Disparities	1	1.6
National Institute of Mental Health	1	1.6
Grant mechanism*		
R01	39	61.9
R21	13	20.6
R03	3	4.8
R37	2	3.2
U01	2	3.2
R15	1	1.6
R34	1	1.6
R56	1	1.6
P01	1	1.6
**Notice of Funding Opportunity (NOFO)**		
Investigator Initiated Funding Opportunity	32	50.8
Targeted Program Announcement or Request forApplications	31	49.2
**Early Stage Investigator (*n* = 43 eligible)**		
Yes	7	16.3
No	36	83.7
**Multiple Principal Investigator Grant**		
Yes	27	42.9

*R01, R21, R03, R37, R15, R34, R56: research grants; U01s: cooperative agreement awards; P01: program project grants

When examining the study population of focus among the funded grants, most (73.0%) included both male and female participants (Table 2). The cancer types most often included in study populations were breast (22.2%), any cancer (17.5%), lung (15.9%), prostate (15.9%), and solid tumors (15.9%). Cancer types less often included were head and neck, brain, bladder, and myeloma (3.2% each), and thymoma (1.6%). Caregivers were included in 15.9% of grants, older adults were the focus of 6.3% of grants, and health care providers (e.g., physician, Advanced Practice Provider) were included in 6.3% of grants (Table 3).

**Table 2:** Study Population (*N* = 63 grants)

Characteristic	*N*	%
**Target population gender**		
Both female and male	46	73.0
Female	12	19.0
Male	5	7.9
Cancer type*		
Breast	14	22.2
Any	11	17.5
Lung	10	15.9
Prostate	10	15.9
Solid tumors	10	15.9
Colorectal	6	9.5
Melanoma	6	9.5
Ovarian	6	9.5
Gastrointestinal	5	7.9
Gynecological	5	7.9
Pancreatic	5	7.9
Esophageal	3	4.8
Head & Neck	2	3.2
Brain	2	3.2
Bladder	2	3.2
Myeloma	2	3.2
Thymoma	1	1.6
Special population*		
Caregivers	10	15.9
Racial and ethnic minority survivors	5	7.9
Older adult survivors	4	6.3
Providers	4	6.3
Other	3	4.8
Rural survivors	2	3.2
Low socioeconomic status survivors	1	1.6

*Check all that apply; will not add up to 100%

**Table 3:** Study Design and Focus (*N* = 63 grants)

Characteristic	*N*	%
**Study Design (*n* = 63)**		
Intervention	34	54.0
Observational	26	41.3
Other	3	4.8
Intervention Design (*n* = 34)*		
Randomized controlled trial	31	91.2
Pilot/feasibility	6	17.6
Single group assignment	3	8.8
Multi-level intervention	1	2.9
**Observational Design (*n* = 26)**		
Cohort/longitudinal (prospective or retrospective)	24	92.3
Cross-sectional	1	3.8
Other	1	3.8
National Cancer Institute Office of Cancer Survivorship Research Focus Area** (*n* = 63)		
Acute psychosocial or physiological toxicity	21	33.3
Late- or long-term effects of cancer and its treatment	21	33.3
Care delivery	14	22.2
Methods	7	11.1
**Specific Study Focus (*n* = 63)**		
Symptom management	14	22.2
Examining physiological or psychological outcomes	9	14.3
Palliative care	8	12.7
Psychosocial support	8	12.7
Healthy behaviors- physical activity, nutrition, weight management	7	11.1
Care delivery	7	11.1
Communication	4	6.3
Predicting adverse events	3	4.8
Role of biomarkers in outcomes	1	1.6
Self-management	1	1.6
Preventing late effects	1	1.6
Cancer Outcomes (primary) (*n* = 63)*		
Psychosocial outcomes	18	28.6
Physiological outcomes	17	27.0
Other***	12	19.0
Healthcare utilization	10	15.9
Health-related quality of life	8	12.7
Biomarkers/biological data	7	11.1
Feasibility/acceptability	7	11.1
Communication; decision-making	7	11.1
Mortality, survival, progression	4	6.3
Models	3	4.8
Clinical outcomes/measures	2	3.2
Health behaviors/health promotion	1	1.6
Accelerated aging	1	1.6
Adherence to treatment	1	1.6

*Check all that apply; will not add up to 100%

**Survivorship research focus area refers to the National Cancer Institute Office of Cancer Survivorship research categories [14]

***Other outcomes include intervention development, values, self-efficacy

Over half of the grants were intervention studies (54.0%), and almost all of these studies were randomized controlled trials (91.2%). We examined the survivorship research focus area based on the categories in the NCI Office of Cancer Survivorship definition of survivorship research [14]. Among all grants, most had a primary focus of acute psychosocial or physiological toxicities (33.3%) or late- or long-term effects of cancer and its treatment (33.3%). Studies also focused on care delivery (22.2%) or methods (11.1%). When examining more specific study focus, 22.2% of all grants focused on symptom management, 14.3% on examining physiological or psychological outcomes for metastatic survivors, 12.7% on palliative care and 12.7% on providing psychosocial support. Primary outcomes of studies included psychosocial (e.g., distress, depression, anxiety) (28.6%), physiological (e.g., pain, fatigue) (27.0%), and healthcare utilization (15.9%).

## Discussion

This paper provides an update to the previously published FY 2020 analysis of NIH grants focused on survivorship research for people living with advanced and metastatic cancers [7]. The previous analysis focused on all grants receiving funding for any year of their grant during FY 2020 and included 25 active grants. In contrast, this analysis examined 63 grants *newly* funded from FY 2021 to FY 2024. Results from this analysis show a substantial increase in funded research in this area. It is notable that half of the grants included were submitted through targeted funding opportunities. Therefore, the growth in the portfolio was not limited to the 8 grants funded by the dedicated RFA on this topic [9]. Other targeted funding opportunities that supported grants in this area were directed to research on cancer caregiving, specific symptoms (e.g., pain), palliative care, and exercise and nutrition.

The dedicated RFA funding opportunity allowed for consideration of studies focused on scientific gaps in this area. The first portfolio analysis identified a need for longitudinal examination of unmet needs, models of care, symptoms related to immunotherapies and other targeted therapies, and financial hardship [7, 8]. Grants funded through the RFA address some of the scientific gaps, including observational studies focused on physiological and psychosocial symptoms experienced by people treated with immunotherapy [15, 16] and with Chimeric Antigen Receptor T-cell (CAR T) therapy [17] and intervention studies focused on strategies to manage symptoms [18, 19] and support psychosocial needs [20]. Outside of the RFA, this analysis revealed further growth in the number of studies examining and addressing the needs of caregivers for people living with advanced and metastatic cancers. Attention to caregivers is critical given the often lengthy trajectory of care that is needed for this population of survivors.

Although included grants address many of the scientific gaps identified in the previous portfolio analysis and the 2021 NCI-led meeting, important areas are still understudied. First, investigators are grappling with effective methods to identify people living with advanced and metastatic cancers in electronic health record (EHR) and registry data [8]. Innovative methods, including the use of artificial intelligence (AI) to proactively identify this population are currently being tested, but more efforts are needed in this area. There is also a persistent need to develop and test models of survivorship care for people living with advanced and metastatic cancers, including the integration of primary care for comorbid conditions that pre-exist or emerge after a cancer diagnosis. Financial hardship and issues with employment for both the survivor and caregiver continue to be lacking among funded studies. With increasing costs of care and the potentially substantial impact of treatment on the ability to work, it is important to identify tailored strategies to support this survivor population. Finally, it is essential to better understand and help mitigate the uncertainty experienced by those impacted by advanced cancer. Research on challenges surrounding coping with uncertainty, as well as prognostic understanding and awareness, including the often-discordant expectations among survivors, caregivers, and providers, is needed.

The results of this portfolio analysis should be viewed in light of certain limitations. First, given the focus on advanced and metastatic cancer survivorship, this analysis does not include grants focused on people with any cancer stage; thus, grants that include but do not exclusively focus on the target population may be excluded. This analysis does not include funding agencies outside of the NIH, and grants funded by the Department of Defense, Patient-Centered Outcomes Research Institute, and other organizations may focus on this population. Additionally, the grants included here were funded through individual grant mechanisms and thus this analysis does not include studies embedded in clinical trials networks (e.g., NCI Community Oncology Research Program). Finally, given constraints with public reporting, it was not possible to include unfunded grant applications in this review. As such, there may be topics that are being proposed but not yet funded that address persistent gap areas and other emerging areas.

### Implications for Cancer Survivors

Survivors living with advanced and metastatic cancers have been strong advocates for research in this area, and the NCI Office of Cancer Survivorship has been committed to listening to their unique needs. This portfolio review highlights key areas where research is needed, but it is critical that studies incorporate survivors as key members of the research team in all phases of the work. Doing so will ensure that we are actively listening and attending to the needs of survivors with direct lived experience.

## Conclusion

Efforts to increase the portfolio focused on survivorship needs for people living with advanced and metastatic cancers have resulted in a substantial increase in the number of newly funded awards between FY 2021 to FY 2024. Several gap areas are beginning to be addressed through the current grants, including longitudinal research focused on survivors treated with newer therapies, and studies focused on cancer caregivers in this context. Notable gap areas include models of survivorship care delivery, financial hardship and employment, and prognostic uncertainty. Overall, given the growth in this population, it will only become more important to fund innovative research to address their needs and ultimately improve their care and outcomes.

## Data Availability

The list of grants that are the focus of this manuscript will be made available upon request by the corresponding author.
